# Supplementary material for: Metagenome-assembled microbial genomes from Parkinson’s disease fecal samples
Source: Sci Rep. 2024 Aug 14;14:18906. doi: 10.1038/s41598-024-69742-4 (PMC11324757; doi:10.1038/s41598-024-69742-4)
Supplement: Supplementary file 10 — Supplementary Information 10. [file 41598_2024_69742_MOESM10_ESM.pdf]

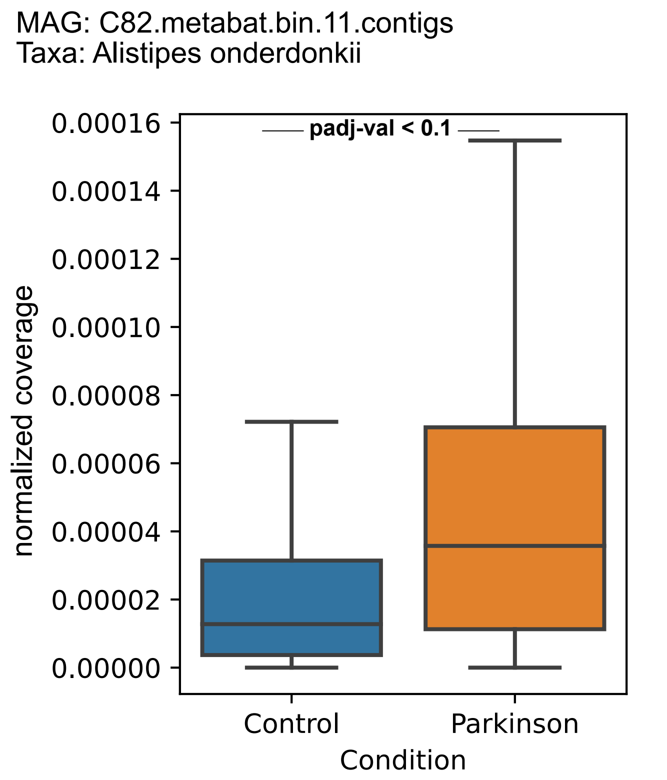
MAG: C82.metabat.bin.11.contigs
Taxa: Alistipes onderdonkii
padj-val < 0.1
normalized coverage
0.00016
0.00014
0.00012
0.00010
0.00008
0.00006
0.00004
0.00002
0.00000
Control
Parkinson
Condition

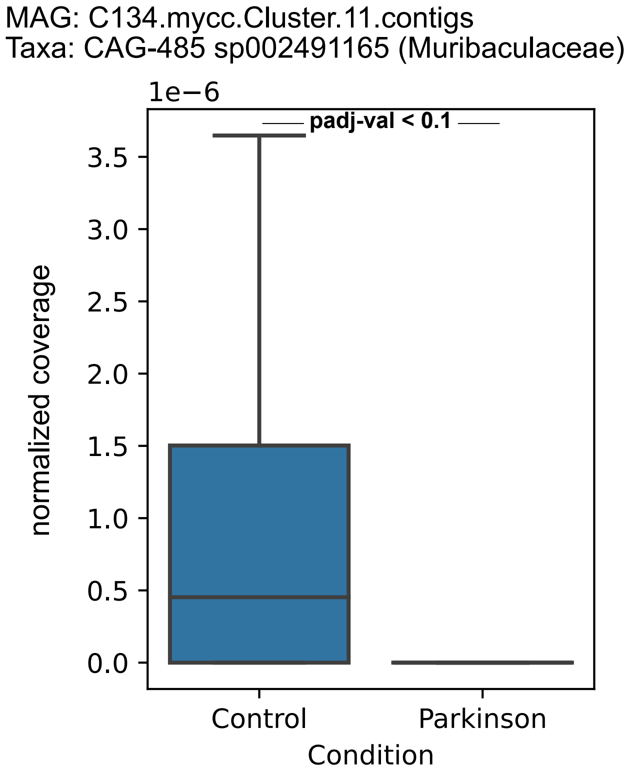
MAG: C134.mycc.Cluster.11.contigs
Taxa: CAG-485 sp002491165 (Muribaculaceae)
1e−6
padj-val < 0.1
normalized coverage
0.0
0.5
1.0
1.5
2.0
2.5
3.0
3.5
Control
Parkinson
Condition

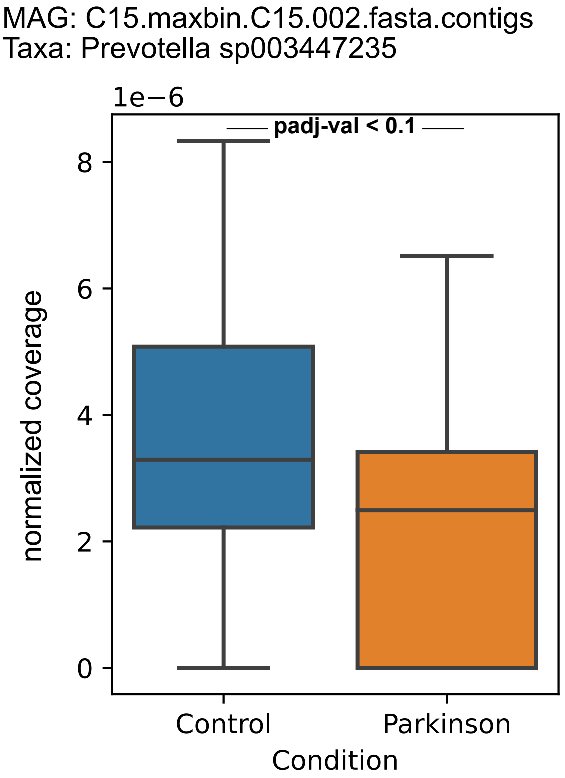
MAG: C15.maxbin.C15.002.fasta.contigs
Taxa: Prevotella sp003447235
1e−6
padj-val < 0.1
8
6
4
2
0
normalized coverage
Control
Parkinson
Condition

MAG: C1.mycc.Cluster.28_sub.contigs
Taxa: Agathobacter sp900549895

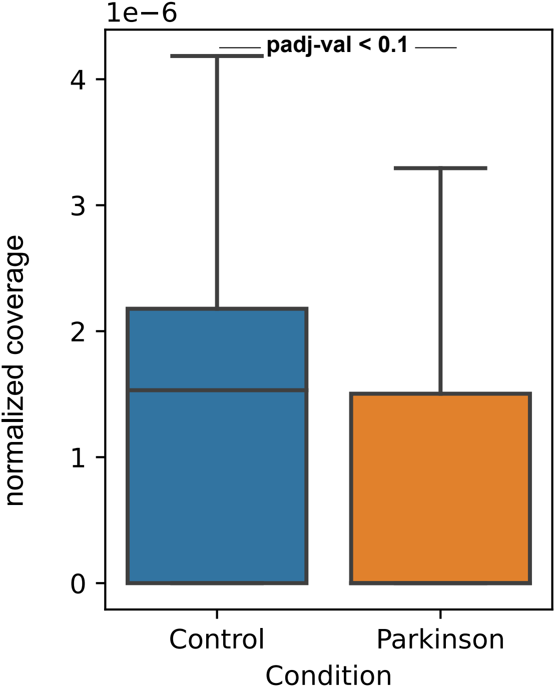

MAG: C33.metabat.bin.54_sub.contigs
Taxa: Agathobacter sp000434275

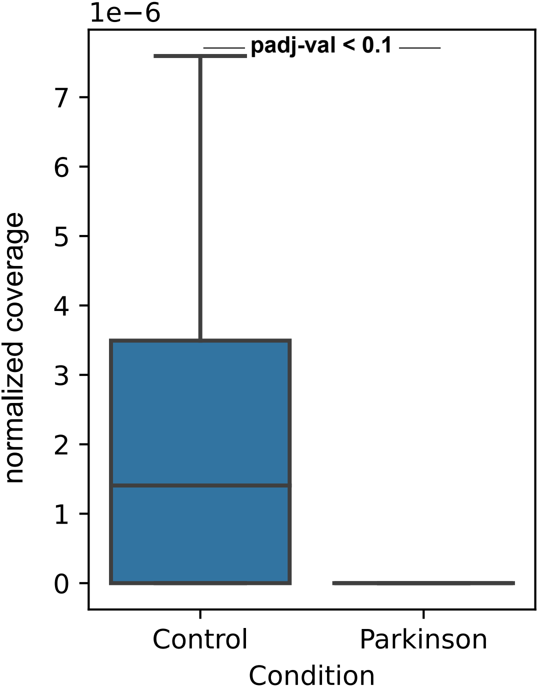

MAG: C70.metabat.bin.84.contigs
Taxa: Dysosmobacter sp900544615

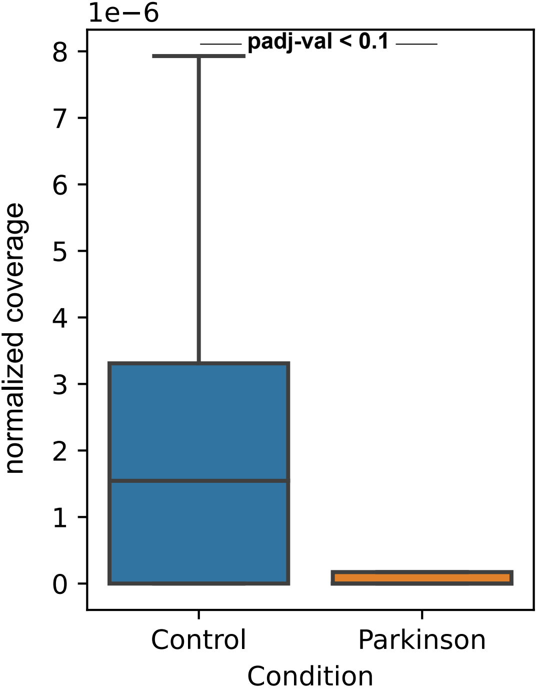

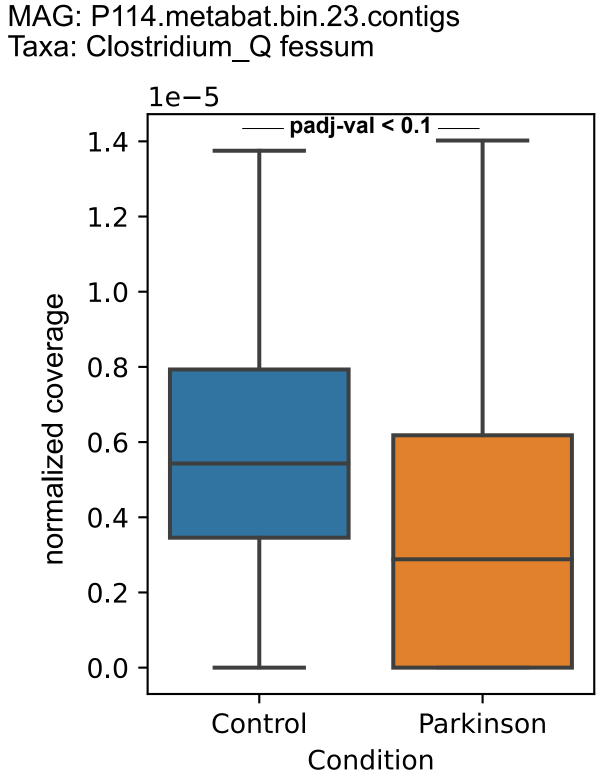

MAG: P114.metabat.bin.23.contigs
Taxa: Clostridium_Q fessum
1e−5
padj-val < 0.1
1.4
1.2
1.0
0.8
0.6
0.4
0.2
0.0
normalized coverage
Control
Parkinson
Condition

MAG: P115.mycc.Cluster.33.contigs
Taxa: Prevotella sp900540415

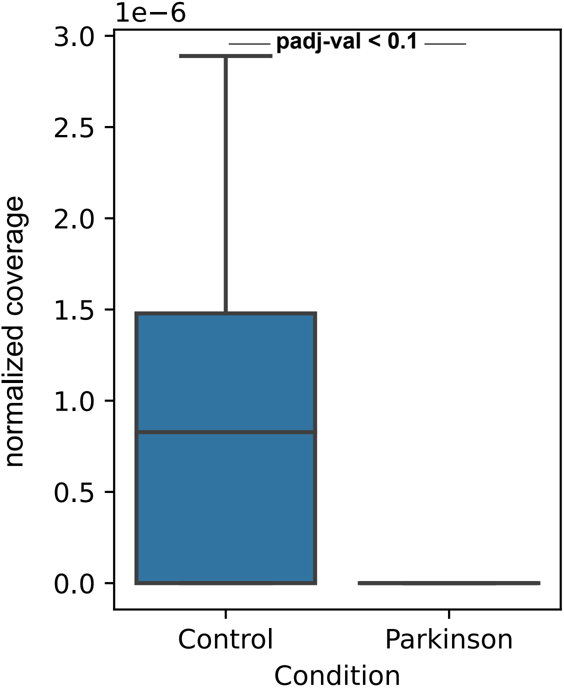

MAG: P16.metabat.bin.41.contigs
Taxa: Choladocola sp018223365

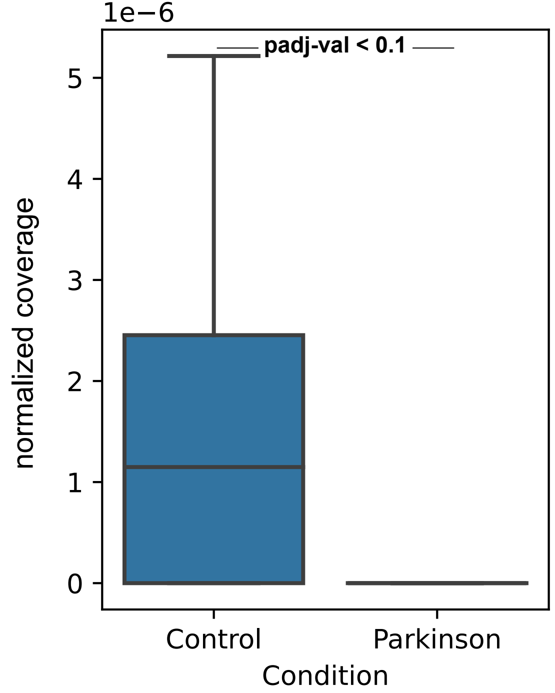

MAG: P66.metabat.bin.18_sub.contigs
Taxa: Blautia_A wexlerae

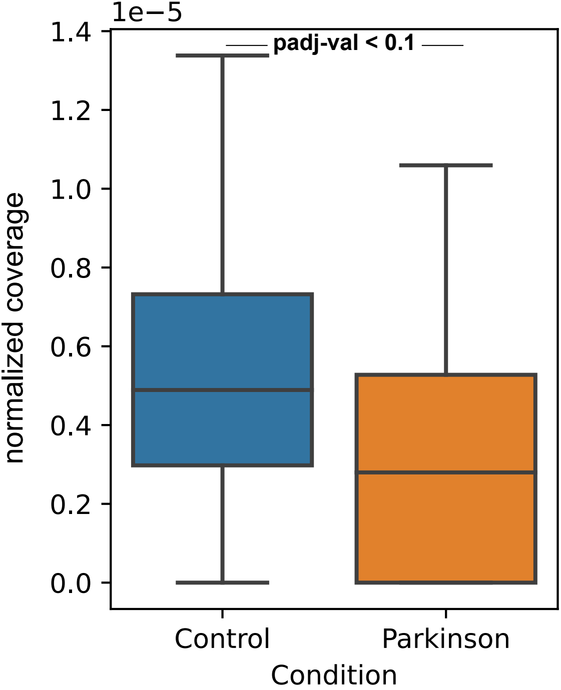

Figure S6. Boxplot of each MAGs that we observed statistically significant difference between two groups in coverage data. The coverage is normalized by the whole sequencing depth. Blue box represent Control samples and orange Parkinson samples. Statistical difference between groups were calculated using Wilcoxon rank-sum statistic with Benjamini/Hochberg correction.
