# Supplementary material for: Metagenome-assembled microbial genomes from Parkinson’s disease fecal samples
Source: Sci Rep. 2024 Aug 14;14:18906. doi: 10.1038/s41598-024-69742-4 (PMC11324757; doi:10.1038/s41598-024-69742-4)
Supplement: Supplementary file 11 — Supplementary Information 11. [file 41598_2024_69742_MOESM11_ESM.pdf]

MAG: C88.maxbin.C88.006.fasta_sub.contigs

GTDB-tk annotation: *Bacteroides eggerthii*

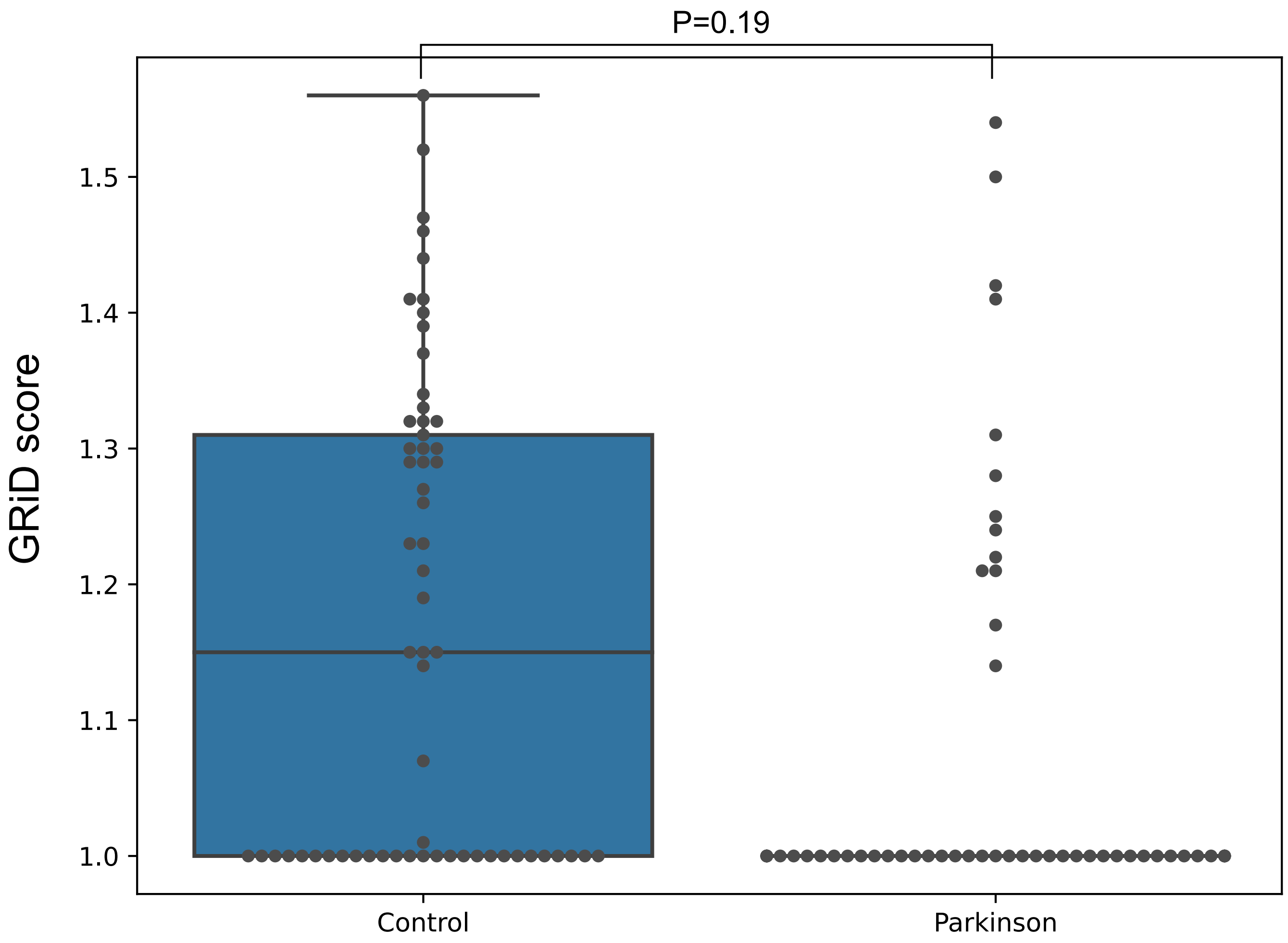

MAG: P73.maxbin.P73.028.fasta_sub.contigs

GTDB-tk annotation: UBA11774 (belongs to Lachnospiraceae family)

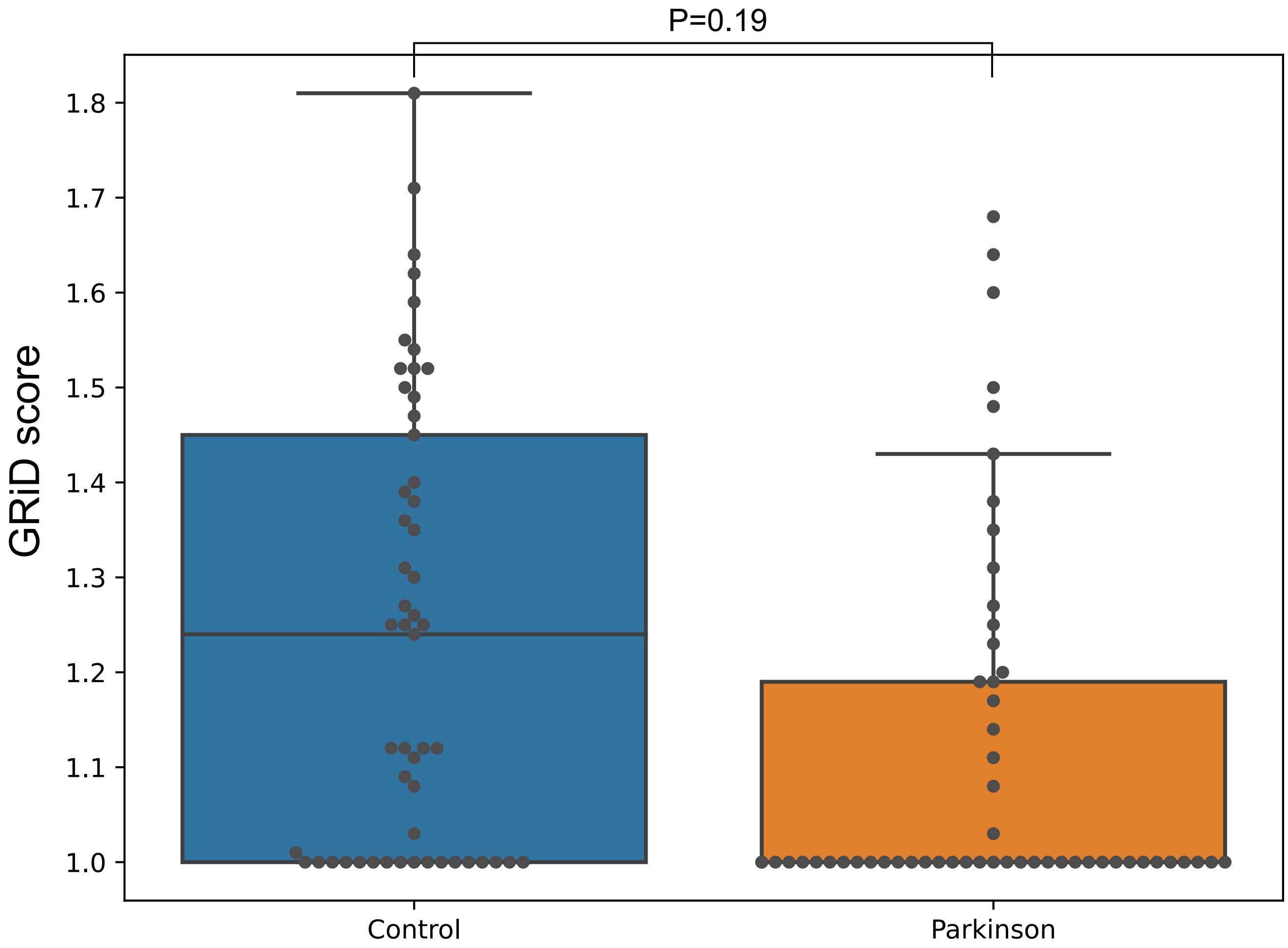

Supplementary Figure S7. Boxplot of each MAGs that we observed difference between two groups with low P-value in Growth Rate Index (GRiD) score. Each dot represents one sample. Blue box represents the Control group, and orange Parkinson. Statistical difference between groups were calculated using Wilcoxon rank-sum statistic with Benjamini/Hochberg correction.
