# Supplementary material for: Metagenome-assembled microbial genomes from Parkinson’s disease fecal samples
Source: Sci Rep. 2024 Aug 14;14:18906. doi: 10.1038/s41598-024-69742-4 (PMC11324757; doi:10.1038/s41598-024-69742-4)
Supplement: Supplementary file 12 — Supplementary Information 12. [file 41598_2024_69742_MOESM12_ESM.pdf]

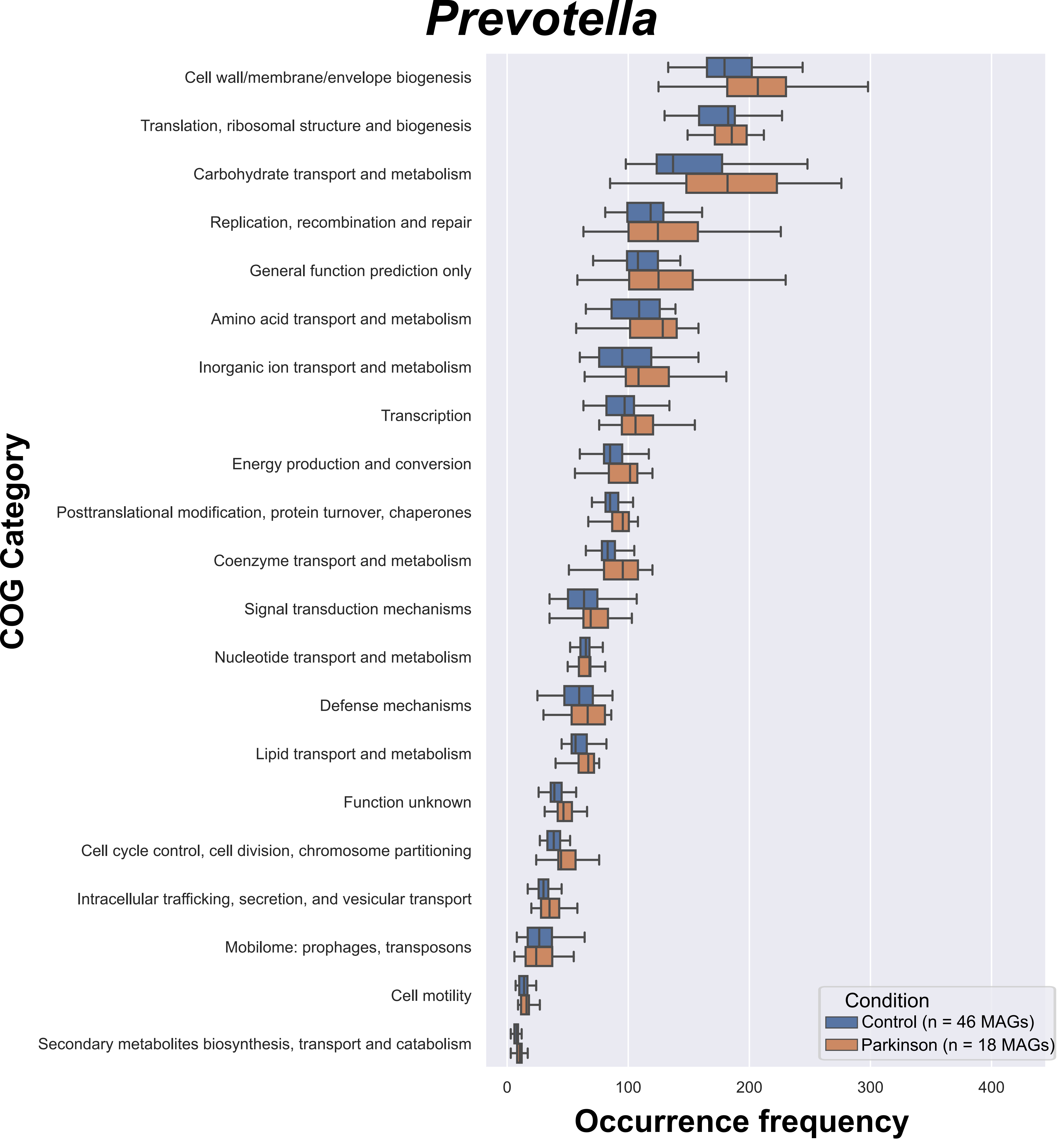

Figure S8. The occurrence frequencies of COG categories in Prevotella MAGs. Each COG category is represented on y axis, and box plot represents the occurrence frequency within the MAGs. Blue is control MAGs, and orange is Parkinson MAGs.
