# Supplementary material for: Metagenome-assembled microbial genomes from Parkinson’s disease fecal samples
Source: Sci Rep. 2024 Aug 14;14:18906. doi: 10.1038/s41598-024-69742-4 (PMC11324757; doi:10.1038/s41598-024-69742-4)
Supplement: Supplementary file 14 — Supplementary Information 14. [file 41598_2024_69742_MOESM14_ESM.pdf]

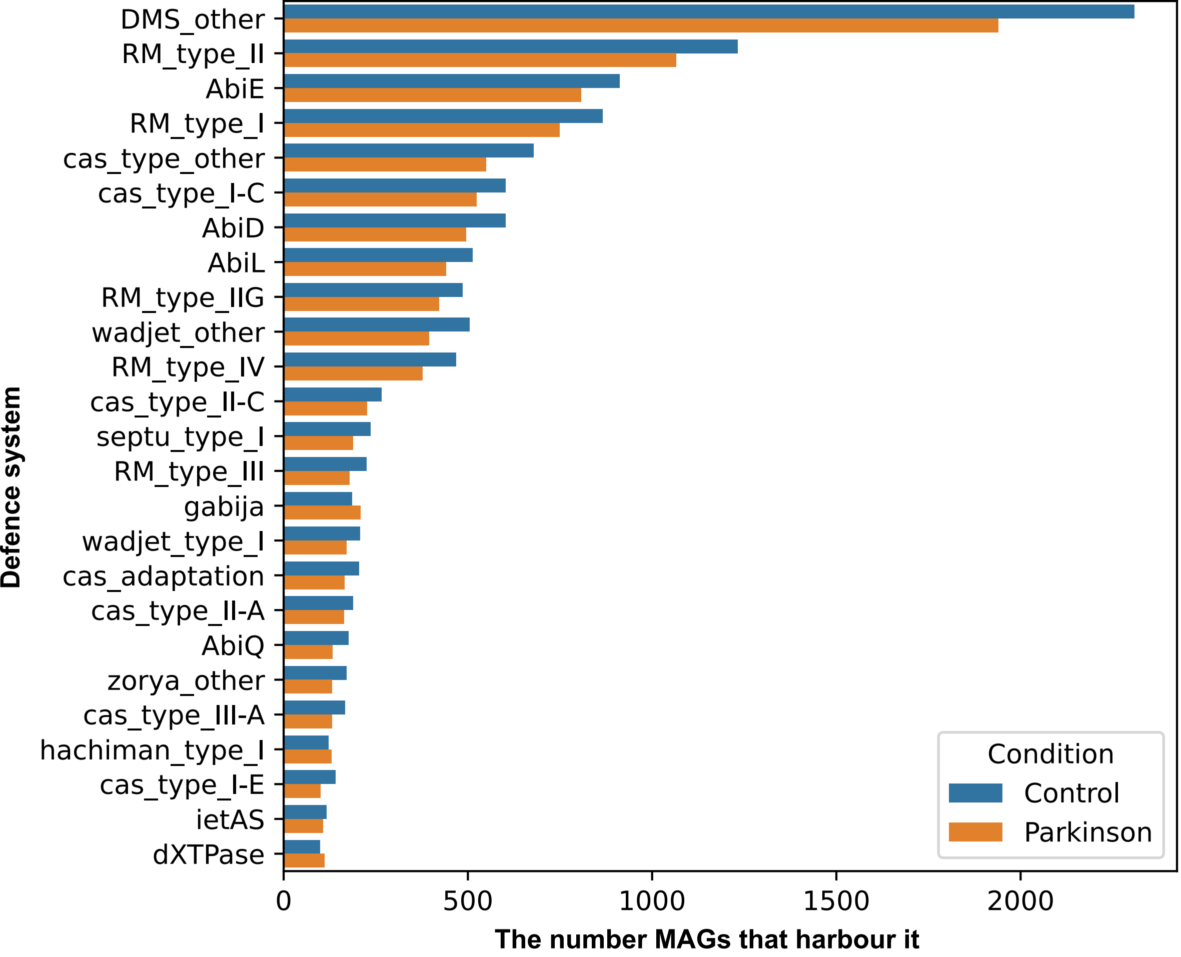

Figure S10. The bar chart shows top 25 defence systems predicted and number of the MAGs that harbour it. Defence system namings are from padlocdb (https://padloc.otago.ac.nz/padloc/systeminformation/). DMS_other represents multiple possible defence systems.

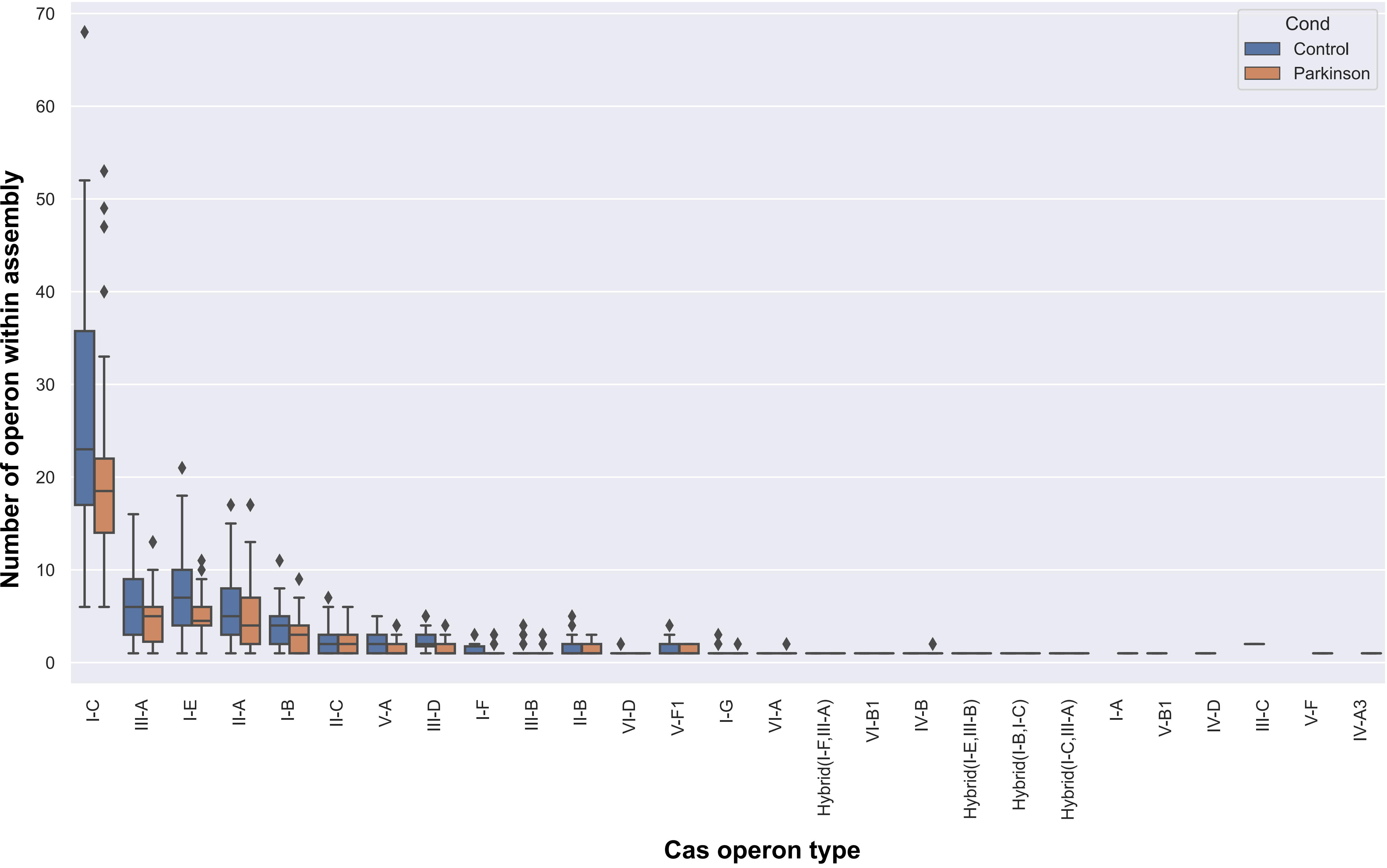

Figure S11.The number of cas operons in each assembly are shown with box plot.
