# Supplementary material for: Metagenome-assembled microbial genomes from Parkinson’s disease fecal samples
Source: Sci Rep. 2024 Aug 14;14:18906. doi: 10.1038/s41598-024-69742-4 (PMC11324757; doi:10.1038/s41598-024-69742-4)
Supplement: Supplementary file 17 — Supplementary Information 17. [file 41598_2024_69742_MOESM17_ESM.pdf]

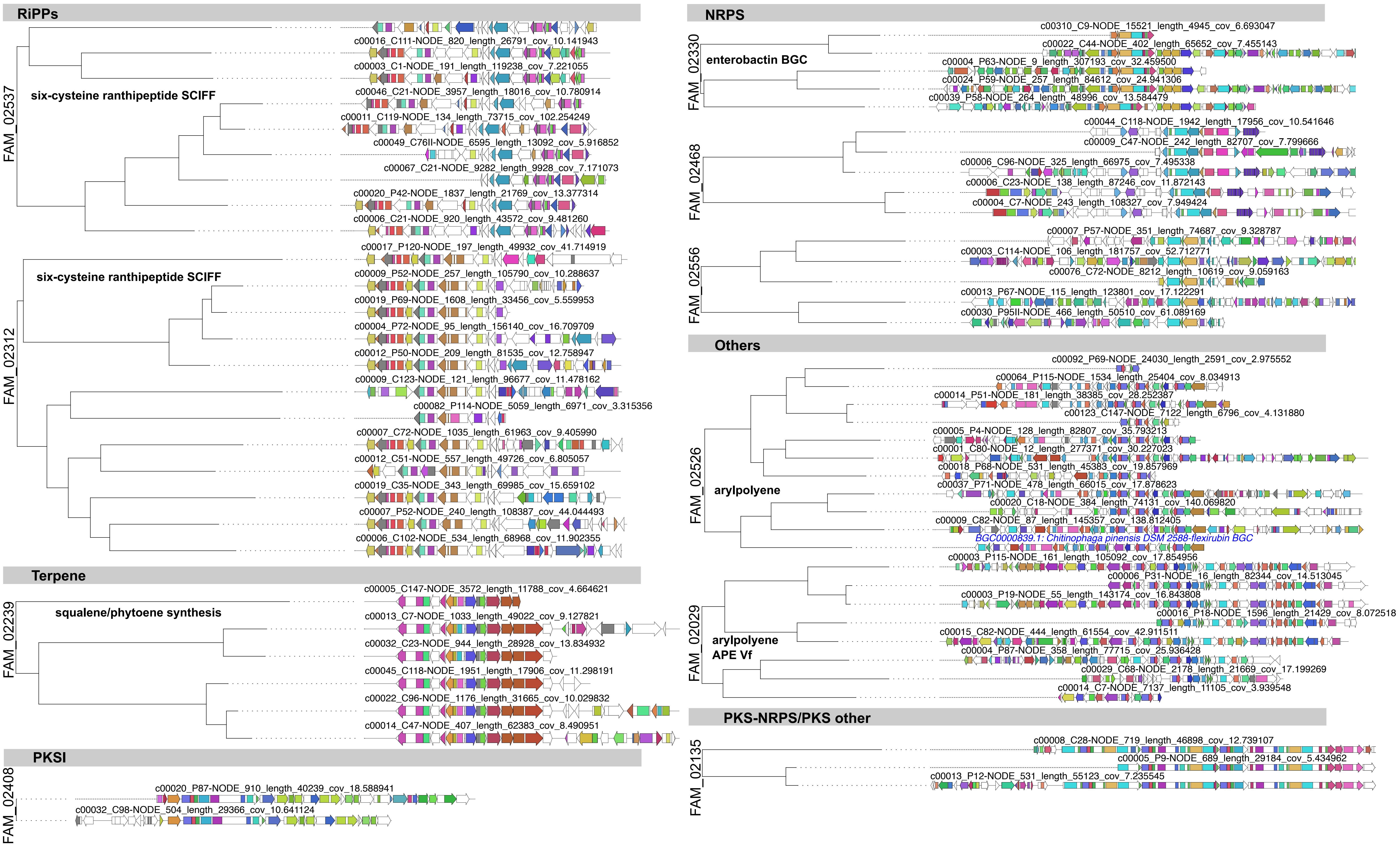

RiPPs
FAM_02537
six-cysteine ranthipeptide SCIFF
c00016_C111-NODE_820_length_26791_cov_10.141943
c00003_C1-NODE_191_length_119238_cov_7.221055
c00046_C21-NODE_3957_length_18016_cov_10.780914
c00011_C119-NODE_134_length_73715_cov_102.254249
c00049_C76II-NODE_6595_length_13092_cov_5.916852
c00067_C21-NODE_9282_length_9928_cov_7.171073
c00020_P42-NODE_1837_length_21769_cov_13.377314
c00006_C21-NODE_920_length_43572_cov_9.481260
FAM_02312
six-cysteine ranthipeptide SCIFF
c00017_P120-NODE_197_length_49932_cov_41.714919
c00009_P52-NODE_257_length_105790_cov_10.288637
c00019_P69-NODE_1608_length_33456_cov_5.559953
c00004_P72-NODE_95_length_156140_cov_16.709709
c00012_P50-NODE_209_length_81535_cov_12.758947
c00009_C123-NODE_121_length_96677_cov_11.478162
c00082_P114-NODE_5059_length_6971_cov_3.315356
c00007_C72-NODE_1035_length_61963_cov_9.405990
c00012_C51-NODE_557_length_49726_cov_6.805057
c00019_C35-NODE_343_length_69985_cov_15.659102
c00007_P52-NODE_240_length_108387_cov_44.044493
c00006_C102-NODE_534_length_68968_cov_11.902355
Terpene
FAM_02239
squalene/phytoene synthesis
c00005_C147-NODE_3572_length_11788_cov_4.664621
c00013_C7-NODE_1033_length_49022_cov_9.127821
c00032_C23-NODE_944_length_28928_cov_13.834932
c00045_C118-NODE_1951_length_17906_cov_11.298191
c00022_C96-NODE_1176_length_31665_cov_10.029832
c00014_C47-NODE_407_length_62383_cov_8.490951
PKSI
FAM_02408
c00020_P87-NODE_910_length_40239_cov_18.588941
c00032_C98-NODE_504_length_29366_cov_10.641124
NRPS
FAM_02330
enterobactin BGC
c00310_C9-NODE_15521_length_4945_cov_6.693047
c00022_C44-NODE_402_length_65652_cov_7.455143
c00004_P63-NODE_9_length_307193_cov_32.459500
c00024_P59-NODE_257_length_84612_cov_24.941306
c00039_P58-NODE_264_length_48996_cov_13.584479
FAM_02468
c00044_C118-NODE_1942_length_17956_cov_10.541646
c00009_C47-NODE_242_length_82707_cov_7.799666
c00006_C96-NODE_325_length_66975_cov_7.495338
c00006_C23-NODE_138_length_87246_cov_11.872143
c00004_C7-NODE_243_length_108327_cov_7.949424
FAM_02556
c00007_P57-NODE_351_length_74687_cov_9.328787
c00003_C114-NODE_106_length_181757_cov_52.712771
c00076_C72-NODE_8212_length_10619_cov_9.059163
c00013_P67-NODE_115_length_123801_cov_17.122291
c00030_P95II-NODE_466_length_50510_cov_61.089169
Others
FAM_02526
arylpolyene
c00092_P69-NODE_24030_length_2591_cov_2.975552
c00064_P115-NODE_1534_length_25404_cov_8.034913
c00014_P51-NODE_181_length_38385_cov_28.252387
c00123_C147-NODE_7122_length_6796_cov_4.131880
c00005_P4-NODE_128_length_82807_cov_35.793213
c00001_C80-NODE_12_length_277371_cov_30.227023
c00018_P68-NODE_531_length_45383_cov_19.857969
c00037_P71-NODE_478_length_66015_cov_17.878623
c00020_C18-NODE_384_length_74131_cov_140.069820
c00009_C82-NODE_87_length_145357_cov_138.812405
BGC0000839.1: Chitinophaga pinensis DSM 2588-flexirubin BGC
FAM_02029
arylpolyene
APE Vf
c00003_P115-NODE_161_length_105092_cov_17.854956
c00006_P31-NODE_16_length_82344_cov_14.513045
c00003_P19-NODE_55_length_143174_cov_16.843808
c00016_P18-NODE_1596_length_21429_cov_8.072518
c00015_C82-NODE_444_length_61554_cov_42.911511
c00004_P87-NODE_358_length_77715_cov_25.936428
c00029_C68-NODE_2178_length_21669_cov_17.199269
c00014_C7-NODE_7137_length_11105_cov_3.939548
PKS-NRPS/PKS other
FAM_02135
c00008_C28-NODE_719_length_46898_cov_12.739107
c00005_P9-NODE_689_length_29184_cov_5.434962
c00013_P12-NODE_531_length_55123_cov_7.235545

Figure S14. Most commonly secondary metabolites BGCs present in the dereplicated MAGs.
